# Diurnal asymmetric warming promotes the growth of the perennial species *Sophora alopecuroides* in a temperate arid region of China

**DOI:** 10.3389/fpls.2026.1796960

**Published:** 2026-06-10

**Authors:** Ying Liu, Hongmei Zhao, Yijun Bai, Mingxia Liu, Gan Zhou, Tiancui Shang, Yu Zhao

**Affiliations:** 1 School of Chemistry and Environmental Science, Shangrao Normal University, Shangrao, China; 2 School of Biological Science and Technology, Yili Normal University, Yining, Xinjiang, China; 3 School of Culture and Tourism Industry, Shangrao Normal University, Shangrao, China; 4 Key Laboratory of Plant Resources Protection and Utilization in Xinjiang Yili Valley, Yining, Xinjiang, China

**Keywords:** asymmetric warming, biomass allocation, functional traits, physiochemical indicators, *Sophora alopecuroides*

## Abstract

**Introduction:**

Global warming exhibits asymmetric patterns (differential day–night warming rates), yet its effects on early plant regeneration and physiological adaptation remain unclear. This study aimed to investigate the effects of different warming regimes on seedling emergence, growth, and physiological traits of *Sophora alopecuroides*, in order to assess its invasive potential under climate warming scenarios.

**Methods:**

A controlled experiment was conducted with symmetric warming, daytime warming (asymmetric), nocturnal warming (asymmetric), and a control treatment. Measurements included seedling emergence time and final emergence percentage, morphological traits (plant height, root length, leaf area), biomass allocation (stem dry mass, root fresh mass, root–shoot ratio, specific root length, specific leaf area), and physiological indices (antioxidant system activity).

**Results:**

Emergence: Symmetric warming significantly advanced the onset of emergence, but none of the warming treatments altered final emergence relative to the control. Growth: Asymmetric warming induced stronger stimulation of leaf area than nocturnal warming; excessively high night-time temperatures suppressed plant height and markedly inhibited root elongation while reducing leaf area. Symmetric warming significantly increased stem dry mass, whereas asymmetric warming produced the highest root fresh mass among all treatments. Biomass allocation: Diurnal asymmetric warming markedly shifted biomass allocation patterns and morphological characteristics; it inhibited root elongation while increasing root biomass accumulation, resulting in an elevated root–shoot ratio but reduced specific root length and specific leaf area. Physiological response: Under diurnal asymmetric warming, plants activated their antioxidant systems to mitigate oxidative damage.

**Discussion:**

Asymmetric warming (particularly diurnal asymmetry) may enhance the invasive potential of *S. alopecuroides* by promoting biomass accumulation and altering resource allocation strategies (e.g., increased root–shoot ratio and activation of antioxidant defense). These adaptive mechanisms might facilitate its rapid spread in the region under climate change scenarios.

## 1 Introduction

The effects of human activities on environments, including global warming, biological invasions, and atmospheric pollution are becoming increasingly significant (Santos and Bakhshoodeh, 2021). In order to more accurately predict the consequences of these activities, a deeper understanding of the impacts on ecosystems is needed, particularly the impacts on plant growth caused by increased Earth’s surface temperature (Alward, 1999; Cunze et al., 2013). Warming has been shown to promote plant growth and development, but it can also inhibit plant growth (Shah and Paulsen, 2003). In changing environments, invasive plants are often better adapted and more tolerant due to their ecophysiological traits, release from natural enemies, and allelopathic effects. An increase of just 2 °C in global temperatures could promote photosynthesis and growth of plants under non-stress conditions, while also promoting interspecific interactions and ecological consequences (Martinez et al., 2014). *Alternanthera philoxeroides* exhibited greater competitiveness than the native species *Digitaria sanguinalis* under warming conditions, with its invasiveness increasing along latitudinal gradients (Wu et al., 2017). Therefore, under global warming environments, even small temperature increases may give invasive species a significant competitive advantage, which could alter the competitive patterns between invasive and native species (Alpert et al., 2000).

Global warming is one of the key features of climate change. However, the warming is not uniform throughout the day and night, and the nighttime warming is more significant than that of the daytime (Zhong et al., 2023). This diurnal warming pattern is known as ‘asymmetric warming’ (Karl et al., 1993; Peng et al., 2013). Over the past half-century, the rate of warming at night is about 1.4 times faster than during the day (Peng et al., 2013). Previous studies mainly focused on changes in daily average or daytime temperatures, and the phenomena of diurnal or seasonal asymmetrical warming have not received sufficient attention. Asymmetric warming in the real environment is often overlooked and even has misleading or opposite effects (Wang et al., 2017). The results of controlled experiments (Wan et al., 2009), remote sensing observations (Peng et al., 2013; Zhu et al., 2022), and model simulations (Rosenzweig and Tubiello, 1996; Dhakhwa and Campbell, 1998) all indicate that the impact of asymmetric warming on different terrestrial ecosystems varies, and there are also some differences between species. For example, asymmetric warming promoted the growth and carbon sequestration capacity of certain tree species in humid areas, while those species in the arid areas are inhibited (Gao et al., 2022). Therefore, to address the challenges posed by climate change, adaptive strategies are needed across sectors affected by diurnal temperature variations, including agricultural production and management of invasive species. Studies have found that asymmetric warming may differentially affect invasive and native plant species, potentially altering invasion dynamics (He and He, 2020; Chen et al., 2017).

Xinjiang is located in the arid and semi-arid zone of China’s temperate continental climate. The region features complex environmental gradients, distinct climate zones, heterogeneous distributions of water and heat, and characteristic “mountain-oasis-desert” ecosystems. In this region, the daily minimum temperature (Tmin) has increased approximately 1.5 times faster than the daily maximum temperature (Tmax) during growing seasons, indicating a significant asymmetry in diurnal warming patterns. Meanwhile, compared to daytime warming, nighttime warming significantly affects vegetation in coniferous forests, grasslands, and meadows in this area, indicating that the asymmetric warming pattern observed here is broadly consistent with the global trend (Zhao et al., 2017). Due to its distinctive geographical, ecological, and locational characteristics, Xinjiang has emerged as one of the regions in China most severely affected by invasive species in agricultural and forest ecosystems (Liu et al., 2017a). In recent half century, due to climate change, overgrazing, and other anthropogenic pressures, the grasslands in this region have undergone severe degradation, with a sharp decline in grassland productivity. Nearly one-third of the grasslands have been affected by noxious plant species, and *Sophora alopecuroides* L. is one of the representative harmful species. In the past 30 years, the spread of *S. alopecuroides* in the Yili grassland has been accelerating, showing a trend of invasion into high-altitude areas, which poses a serious threat to the livestock production and ecosystem security in this area.

*Sophora alopecuroides* L. is a perennial herbaceous plant of *Sophora (family Fabaceae)*, which has a strong ability to adapt to many kinds of habitats and is commonly found in river valley terraces, deserts and oases in arid and semi-arid areas of China. The high seed yield and strong vegetative reproduction capacity via underground rhizomes jointly contribute to its strong invasiveness and dispersal potential. At the same time, due to the abundance of secondary metabolites with allelopathic effects in the roots, stems, leaves, and seeds of *S. alopecuroides*, it has a significant inhibitory effect on seed germination and seedling growth of various local species (Liu et al., 2017a; Lei et al., 2021), and it holds significant competitive advantages over native grassland species. However, it is still unclear how *S. alopecuroides* respond to the asymmetric warming in this area, and whether it promotes the rapid spread of this local invasive species. If there is a promoting effect, how does this species respond and what are the possible response patterns or mechanisms? Therefore, this study took *S. alopecuroides* as the material and attempted to explore the effect of asymmetric warming on its growth through controlled warming experiments. It also attempted to answer the following three scientific questions: (1) Does asymmetric warming affect the growth of *S. alopecuroides* and what impact does it have on the allocation pattern of biomass? (2) Do the physicochemical characteristics of *S. alopecuroides* respond significantly to asymmetric warming? (3) Does warming, especially asymmetric warming, promote the rapid spread of *S. alopecuroides* in the region? The results of this study will contribute to understanding the ecological mechanisms underlying the rapid expansion of *S. alopecuroides* in this region and provide baseline data for informing grassland ecosystem management strategies.

## 2 Materials and methods

### 2.1 Materials

*S. alopecuroides* L. seeds were collected from mountain steppe in the suburbs of Yining City, Ili Kazakh Autonomous Prefecture, Xinjiang, China. The identification of *S. alopecuroides* L. was conducted by Prof. Yan Ping (College of Life Sciences, Shihezi University) and the herbarium specimen was deposited in the Institute of Botany, Chinese Academy of Sciences (specimen code: PE00214699). This species is widely distributed in northwest China, commonly found in mountainous grasslands and farmland, and is a local invasive species that can be obtained without the need for a permit at the time of sampling.

### 2.2 Experimental methods

*S. alopecuroides* seeds were selected for this study. Mechanical disruption was used to eliminate seeds’ dormancy. Subsequently, the seeds were disinfected with 1% NaClO for 5 min, soaked in distilled water for 24 h at 20 °C. Seeds were sown in plastic pots (21 × 15 × 18 cm) with depth of 1 cm and 15 seeds per pot. Based on the characteristics of climate change in Xinjiang from 1982~2013 (Zhao et al., 2017), the seasonal temperature characteristics of *S. alopecuroides* germination in wild habitats, and the characteristics of global warming, this study set the warming at 3 °C. Each warming treatment’s daytime and nighttime temperatures were set, including a control (25 °C/15 °C, CK), daytime warming (28 °C/15 °C, T1), nighttime warming (25 °C/18 °C, T2), symmetric warming (28 °C/18 °C, T3), and asymmetric warming (28 °C/20 °C, T4). Artificial climate chambers were employed as the warming method. Pots were placed in artificial climate chambers (12-h light/12-h dark, light intensity of 6000 LX), based on our field monitoring, *S. alopecuroides* seedlings exhibited optimal growth within a moderate light range of 3000–9000 LX; therefore, a light intensity of 6000 LX was selected to ensure normal seedling development while maintaining the stability of the warming gradient treatments. Each treatment consisted of 15 pots, for a total of 75 pots.

### 2.3 Sample measurement and related indices evaluation

For each treatment, 30 seedlings with similar growth status were selected to measure 19 functional traits of *S. alopecuroides* leaves and taproots under different day-night warming treatments (**Table 1**). Leaf area was determined by scanning leaves with an EPSON leaf area scanner and analyzing morphological characteristics using Image J software. Leaf, root, and stem fresh weights were measured using an analytical balance, then oven-dried at 80 °C to constant weight and reweighed to obtain dry weights of each component. Leaf mass ratio, root mass ratio, stem mass ratio, and root-shoot ratio were calculated using plant fresh weight. Specific Leaf Area (SLA) and Specific Root Length (SRL) are core functional traits characterizing plant carbon acquisition and resource allocation strategies (Jiang et al., 2021). SLA is defined as the ratio of leaf one-sided area to its oven-dry mass (cm²·g^-^¹), reflecting the investment efficiency of light energy capture; SRL is defined as the total length of roots per unit mass (m·g^-^¹), representing the spatial exploration efficiency of soil resources.

**Table 1:** List of the 19 plant functional traits measured.

Organ	Functional traits	Abbreviation	A formula to calculate
Leaf	Leaf area (cm²)	LA	—
	Specific leaf area	SLA	Leaf area/Leaf dry weight
	Leaf fresh weight (g)	LFW	—
	Leaf dry weight (g)	LDW	—
	Number of compound leaf	NCL	—
	Number of leaves	NL	—
	Leaf biomass ratio	LBR	Leaf biomass/Plant biomass
Stem	Plant height (cm)	PH	—
	Stem fresh weight (g)	SFW	—
	Stem dry weight (g)	SDW	—
	Stem biomass ratio	SBR	Stem biomass/Plant biomass
Root	Root length (cm)	RL	—
	Specific root length	SRL	Root length/Root dry weight
	Root fresh weight (g)	RFW	—
	Root dry weight (g)	RDW	—
	Root biomass ratio	RBR	Root biomass/Plant biomass
	Root shoot ratio	RSR	Root biomass/Above ground biomass
Total	Total fresh weight	TFW	—
	Total dry weight	TDW	—

Seed germination parameters were calculated as follows: Initial emergence period refers to the number of days from sowing to the first seed germination (radicle breaking through seed coat), expressed in days (d); Emergence peak refers to the number of days from sowing to the peak emergence date, expressed in days (d); Emergence rate refers to the percentage of normally emerged seeds to total tested seeds, calculated as EP = Ne/Nt × 100%, where Ne is the number of normally emerged seeds (standard: radicle breaking through seed coat ≥ 2 mm) and Nt is the total number of tested seeds.

Soluble protein content was determined by the Bradford method (Bradford, 1976): 100 μL of sample supernatant was mixed with 5 mL Coomassie Brilliant Blue G-250 staining solution (10% Coomassie Brilliant Blue G-250, 5% ethanol, 10% phosphoric acid), incubated at room temperature for 5 min, and absorbance was measured at 595 nm using bovine serum albumin (BSA) as a standard. Soluble sugar content was determined by anthrone colorimetry (Yemm and Willis, 1954): 200 μL of sample supernatant was mixed with 5 mL anthrone-sulfuric acid reagent (0.2 g anthrone dissolved in 100 mL 80% sulfuric acid), heated in boiling water bath for 10 min, cooled, and absorbance measured at 620 nm using glucose as a standard. Malondialdehyde (MDA) content was determined by thiobarbituric acid (TBA) method (Heath and Packer, 1968): 1.0 mL sample supernatant was mixed with 1.0 mL 20% trichloroacetic acid (TCA) and 1.0 mL 0.6% TBA (prepared with 10% TCA), heated in boiling water bath for 20 min, rapidly cooled, then centrifuged at 4 °C and 8000 × g for 10 min. Absorbance of the supernatant was measured at 532 nm, 600 nm, and 450 nm, and MDA content was calculated using the formula: MDA content = 6.45 (A532 - A600) - 0.56 A450, expressed as nmol·g^-^¹ FW.

Fresh leaves from seedlings of each treatment were collected for determination of SOD, CAT, POD, and GSH enzyme activities (Klaus, 2004). Superoxide dismutase (SOD), peroxidase (POD), catalase (CAT) activities and reduced glutathione (GSH) content were measured using commercial assay kits purchased from Beijing Solarbio Science & Technology Co., Ltd. SOD activity was determined by WST-1 method using SOD Activity Assay Kit (Cat. No. BC5160, 50T/24S) based on WST-1 chromogenic reaction detecting the inhibition of superoxide anion free radicals (O_2_^-^·) by SOD, with absorbance measured at 450 nm. POD activity was determined by visible spectrophotometry using POD Activity Assay Kit (Cat. No. BC0090, 50T/48S), calculated from absorbance changes at 470 nm due to POD-catalyzed oxidation of guaiacol by H_2_O_2_ producing brown products. CAT activity was determined by UV spectrophotometry using CAT Activity Assay Kit (Cat. No. BC0200, 50T/48S), calculated from the decrease in absorbance at 240 nm due to CAT-catalyzed decomposition of H_2_O_2_. GSH content was determined by visible spectrophotometry using GSH Content Assay Kit (Cat. No. BC1170, 50T/48S), based on DTNB reaction with GSH sulfhydryl groups forming yellow complexes, with absorbance measured at 412 nm and content calculated from standard curve. All measurements were performed with five replicates. Enzyme activities were expressed as U·mg^-^¹ protein and GSH content as μg·g^-^¹ FW or μg·mg^-^¹ protein.

### 2.4 Statistical analysis

All experimental data were organized by Excel 2016, and Figures were conducted by Origin 2022 software. Statistical analysis, one-way analysis of variance (ANOVA), Duncan’s multiple comparison tests were conducted by SPSS 26.0. The objective of this study was to ascertain differences in functional traits among different warming treatments. Pearson’s correlation analysis was conducted to investigate the relationships among functional traits.

## 3 Results

### 3.1 Seedlings’ emergence of *S. alopecuroides* under different warming treatments

As shown in **Table 2**, warming treatments significantly affected the initial emergence time of *S. alopecuroides*, whereas neither the emergence peak nor the emergence rate differed significantly among treatments. Compared to the daytime warming treatment (T1), symmetrical warming (T3) significantly shortened the initial emergence time by approximately 0.5 days. The initial emergence time under asymmetric warming (T4) was intermediate at 4.5 days, which did not differ significantly from either T3 or T1. The emergence peak occurred approximately 1–2 days after the initial emergence across all treatments, with no significant differences among warming treatments or relative to the control. Similarly, warming did not significantly affect the final emergence rate of *S. alopecuroides* seedlings under any treatment regime.

**Table 2:** Seed emergence of *S. alopecuroides* seedlings in warming habitats.

Group	Initial emergence period (d)	Emergence peak (d)	Rate of emergence %
CK	4.6 ± 0.507ab	5.9 ± 0.640a	61.3 ± 0.143a
T1	4.8 ± 0.414a	5.7 ± 0.594a	56.9 ± 0.087a
T2	4.5 ± 0.516ab	5.5 ± 0.640a	60.9 ± 0.133a
T3	4.3 ± 0.501b	6.0 ± 1.414a	58.6 ± 0.120a
T4	4.5 ± 0.431ab	5.6 ± 1.254a	59.0 ± 0.130a

Different lowercase letters indicate significant differences among warming treatments (p < 0.05). The same notation applies hereafter.

(mean ± SD).

### 3.2 Seedlings’ growth of *S. alopecuroides* under different warming treatments

As shown in **Table 3**, Seedling height was promoted under all warming treatments, with symmetric warming producing the strongest effect, significantly exceeding that of the control, daytime warming, nighttime warming, and asymmetric warming. The elevated nighttime temperature in the asymmetric treatment appeared to constrain height increase relative to symmetric warming. Daytime warming, symmetric or asymmetric warming did not significantly promote roots’ elongation growth, but nighttime warming significantly inhibited root growth. At the same time, root length of asymmetric warming was significantly shorter than that of symmetric warming treatment, indicating that excessively high nighttime temperatures inhibited root elongation. Compared with the control, warming significantly promoted leaf development, with increased numbers of compound leaves and leaflets. The promoting effects of asymmetric and symmetric warming on leaf development were significantly stronger than those of daytime or nighttime warming alone, suggesting that combined day-night warming regimes were more favorable for leaf development. Daytime, symmetric, and asymmetric warming all significantly promoted leaf area expansion, whereas nighttime warming significantly reduced leaf area, indicating that elevated nighttime temperatures were detrimental to leaf growth in *S. alopecuroides*. .

**Table 3:** Morphological characteristics of *S. alopecuroides* seedlings in warming habitats.

Group	Plant height (cm)	Root length (cm)	Number of compound leaves	Number of leaflets	Leaves area (cm²)
CK	13.956 ± 0.254d	12.823 ± 0.472ab	4.100 ± 0.055d	11.400 ± 0.316d	7.728 ± 0.224c
T1	15.206 ± 0.286bc	13.283 ± 0.443ab	4.700 ± 0.085c	13.566 ± 0.354c	9.164 ± 0.283b
T2	15.930 ± 0.330b	10.623 ± 0.360c	4.733 ± 0.095c	13.500 ± 0.358c	6.301 ± 0.233d
T3	17.950 ± 0.380a	14.153 ± 0.657a	5.166 ± 0.108b	15.033 ± 0.353b	10.179 ± 0.307a
T4	14.758 ± 0.306cd	12.344 ± 0.293b	5.733 ± 0.135a	18.500 ± 0.535a	8.179 ± 0.349c

(mean ± SD).

### 3.3 Biomass allocation of *S. alopecuroides* seedlings under different warming treatments

Stem dry biomass under symmetric warming reached 0.029 g, significantly exceeding that of the control, daytime warming, nighttime and asymmetric warming (**Table 4**). The fresh biomass of leaves under daytime warming reached 0.144 g, which was significantly higher than that of the asymmetric treatment (0.128 g). However, the dry biomass of roots under asymmetric warming (0.019 g) was significantly higher than that under daytime warming (0.013 g). For the fresh biomass of stems, there was no significant difference between symmetrical warming and daytime warming, but they were significantly higher than the control. Compared with asymmetric warming, root fresh biomass under all other warming treatments was significantly lower, with the ranking: symmetric warming > daytime warming > nighttime warming.

**Table 4:** Biomass accumulation of *S. alopecuroides* seedlings in warming habitats.

Group	SFW (g)	LFW (g)	RFW (g)	TFW	SDW (g)	LDW (g)	RDW (g)	thW
CK	0.079 ± 0.002b	0.122 ± 0.004bc	0.088 ± 0.006b	0.288 ± 0.01c	0.024 ± 0.001c	0.024 ± 0.001b	0.013 ± 0.001b	0.057 ± 0.002cd
T1	0.096 ± 0.003a	0.144 ± 0.004b	0.091 ± 0.005b	0.331 ± 0.009ab	0.019 ± 0.001b	0.028 ± 0.001a	0.013 ± 0.001b	0.065 ± 0.002bc
T2	0.084 ± 0.002b	0.110 ± 0.003d	0.081 ± 0.003b	0.275 ± 0.006c	0.027 ± 0.001c	0.020 ± 0.001b	0.009 ± 0.001c	0.048 ± 0.001d
T3	0.103 ± 0.002a	0.160 ± 0.006a	0.100 ± 0.007b	0.363 ± 0.012a	0.029 ± 0.001a	0.030 ± 0.001a	0.015 ± 0.001b	0.072 ± 0.003a
T4	0.051 ± 0.002c	0.128 ± 0.005c	0.131 ± 0.010a	0.309 ± 0.015bc	0.021 ± 0.001d	0.032 ± 0.001a	0.019 ± 0.001a	0.067 ± 0.005b

(mean ± SD).

**Figure 1:**
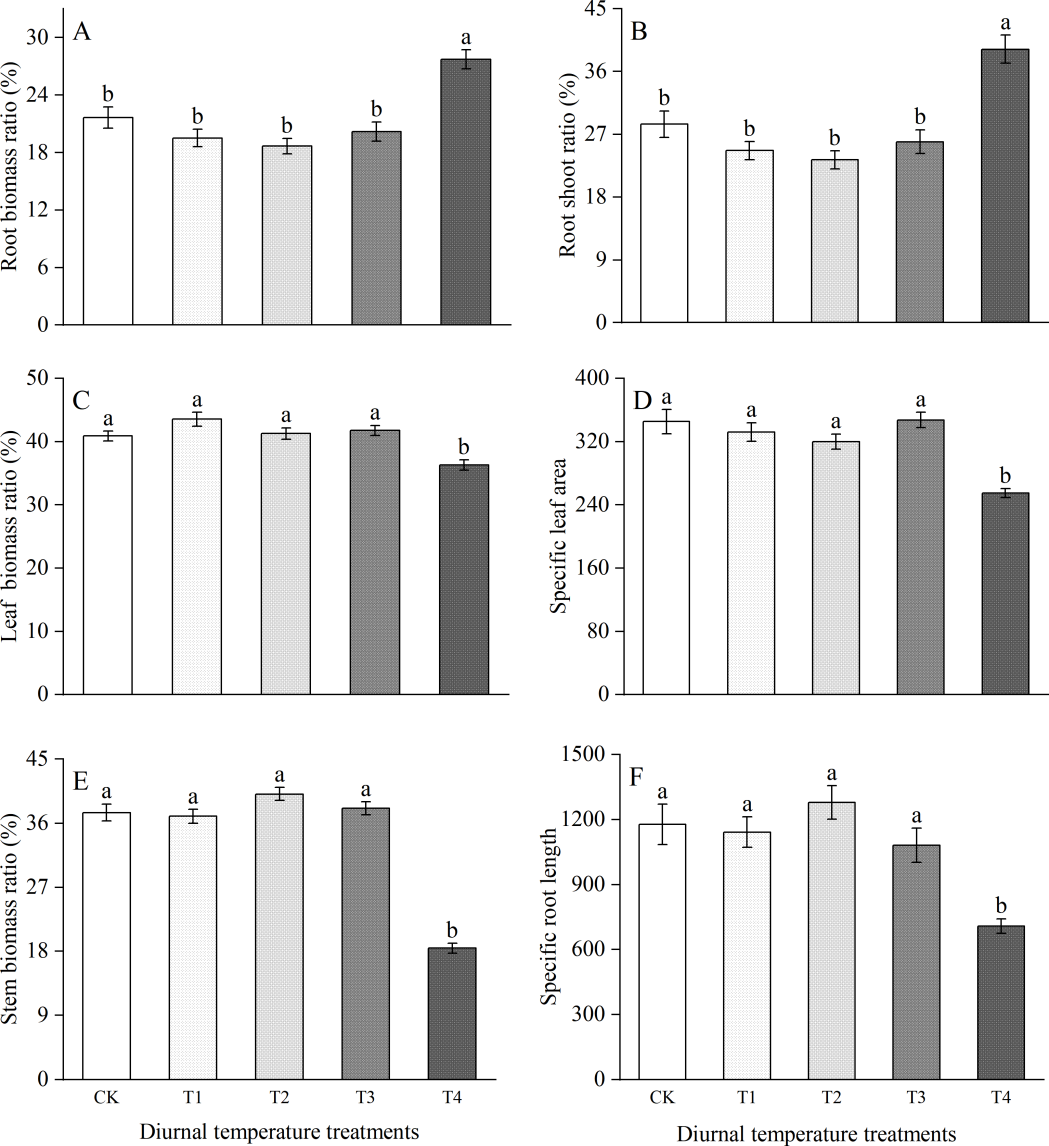
Effects of different diurnal temperature treatments (CK, T1, T2, T3, T4) on plant growth parameters. **(A)** Root biomass ratio, **(B)** Root-to-shoot ratio, **(C)** Leaf biomass ratio, **(D)** Specific leaf area, **(E)** Stem biomass ratio, **(F)** Specific root length.

Under asymmetric warming treatment, the biomass ratio of leaves was significantly higher than that of other warming treatments and the control, whereas the biomass ratio of stems was significantly lower (**Figures 1A, C, E**). Notably, the root biomass ratio was also higher under asymmetric warming, with the root-shoot ratio reaching 0.392, significantly exceeding that of the control and other warming treatments (**Figure 1B**). The specific leaf area (SLA) and specific root length (SRL) of asymmetric treatment were significantly lower than those of control group and other warming treatments (**Figures 1D, F**). Except for asymmetric warming, there were no significant changes in the biomass ratio, SLA and SRL of *S. alopecuroides* seedlings between the control and other warming treatments, or among the other three warming treatments. These findings meant that asymmetrical warming has a significant effect on the biomass allocation of *S. alopecuroides* seedlings.

### 3.4 Physicochemical activities of *S. alopecuroides* seedlings under different warming treatments

**Figure 2:**
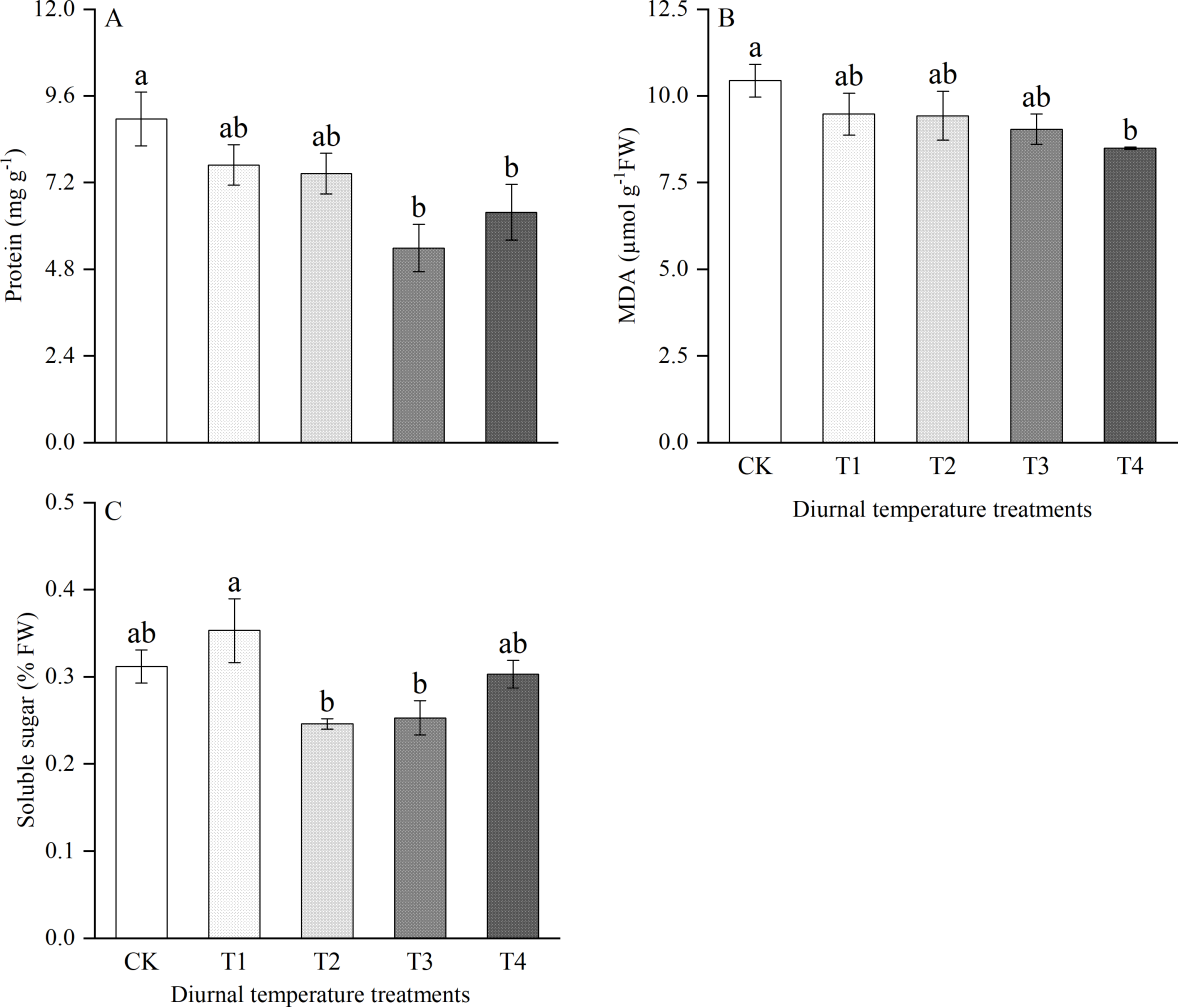
Effects of different diurnal temperature treatments (CK, T1, T2, T3, T4) on protein content, MDA content, and soluble sugar content. **(A)** Protein. **(B)** MDA. **(C)** Soluble sugar.

Under asymmetric and symmetrical warming treatments, the contents of soluble protein reached 5.387 mg/g and 6.377 mg/g, respectively, which were significantly lower than that of the control treatment (**Figure 2A**). Although there was no significant difference in protein content between the daytime or nighttime warming treatment and the control treatment, these results suggested that the increase in temperature may lead to a certain degree of inhibition of soluble protein synthesis. The MDA content of seedlings in the asymmetric treatment was significantly lower than that of the control, but there was no significant difference compared to other warming treatments. Although other warming treatments were also lower than the control, the difference did not reach a significant level (**Figure 2B**). The content of soluble sugars showed no significant changes under various warming treatments. Compared to the control, there was no significant change in the content of soluble sugars in the seedlings of each warming treatment (**Figure 2C**). However, the soluble sugar in the nighttime warming and symmetrical warming treatments were significantly lower than that in the daytime warming treatment.

**Figure 3:**
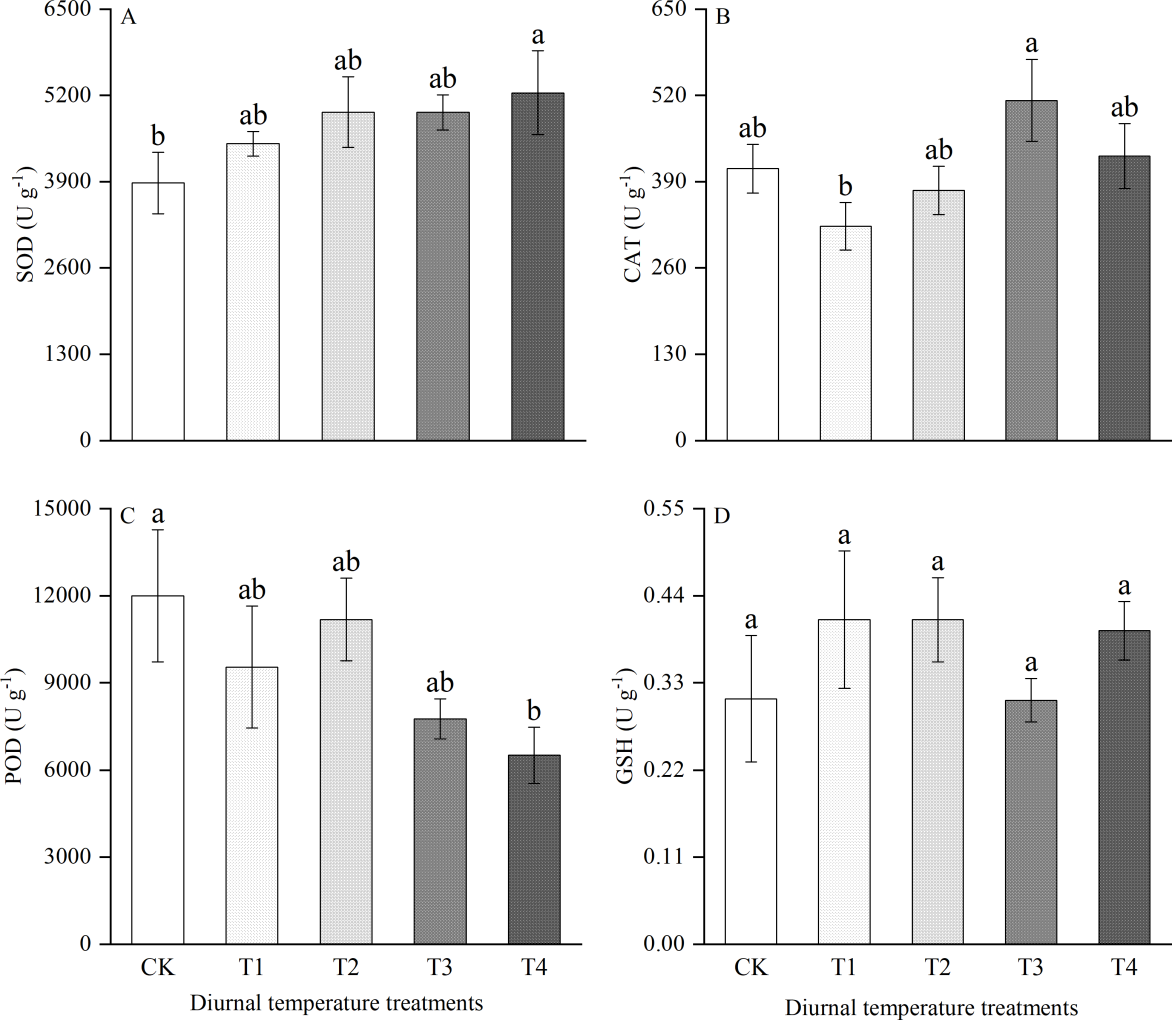
Effects of different diurnal temperature treatments (CK, T1, T2, T3, T4) on the activities of SOD, CAT, POD, and GSH enzymes. **(A)** SOD, **(B)** CAT, **(C)** POD, **(D)** GSH.

**Figure 3** showed the changes in the activities of superoxide dismutase (SOD), catalase (CAT), peroxidase (POD) and glutathione (GSH) content in *S. alopecuroides* seedlings under different warming modes. All warming treatments increased the SOD activity of seedlings, showing T4>T3>T2>T1>CK, and the asymmetric warming was significantly higher than the control (**Figure 3A**). The POD activity of each warming treatment decreased to varying degrees, but only the asymmetric warming showed a significant decreased (**Figure 3C**). The activity of CAT under daytime warming treatment reached 322.728 U/g, which was significantly lower than that of the symmetrical warming, but there was no significant difference compared to other treatment groups (**Figure 3B**). Compared with the control, except for the symmetrical warming, the GSH content of seedlings in other warming treatments increased to varying degrees, but the increase was not significant (**Figure 3D**).

## 4 Discussion

Photosynthesis is one of the key processes in ecosystems, and temperature is one of the primary controlling factors. With the intensification of global climate warming, the responses of plants to different temperature changes have become a research hot spot. This study found that daytime warming, symmetric day-night warming, and asymmetric warming promoted the total biomass accumulation of *Sophora alopecuroides*, whereas nighttime warming alone exhibited significant inhibitory effects. These results differed from those reported by Barton and Schmitz (2017) in a grassland ecosystem, where daytime warming enhanced plant diversity by altering predator-prey spatial distribution, while nighttime warming reduced plant diversity due to increased predation risk. This discrepancy suggested that the effects of diurnal warming patterns on plant growth may vary depending on ecosystem type and species-specific traits (Xia et al., 2018; Yuan et al., 2025). The promotion effect of symmetrical warming on the biomass of *S. alopecuroides* was manifested through the increase of aboveground (stem) biomass, whereas asymmetric warming primarily enhanced belowground (root) biomass accumulation. The enhanced root biomass under warming regimes involving nighttime temperature elevation was broadly consistent with the findings of Wang et al. (2020), who reported that nighttime warming enhances ecosystem carbon-use efficiency in temperate steppes. The results of this study indicated that the promoting effects of day-night asymmetric warming on leaf development were manifested through a significant increase in the number of compound leaves and leaflets, a pattern consistent with the phenomenon of temperature-induced increases in total leaf area (Wei et al., 2023). This result suggested that in resource acquisition strategies, *S. alopecuroides* might compensate for the inhibitory effect of nighttime high temperatures on individual leaf area by increasing the number of photosynthetic organs. Under symmetric warming conditions, plants achieved more efficient biomass allocation by synchronously optimizing their aboveground resource acquisition strategies and underground resource exploration capacity. These findings aligned with the optimal partitioning theory, which posits that plants allocate biomass to organs that acquire the most limiting resources (Poorter and Nagel, 2000).

The differences in the effects of day-night warming on the biomass of *S. alopecuroides* were closely related to the “source-sink” relationship of plant photosynthesis. Daytime warming promoted the accumulation of organic matter by increasing photosynthetic rates and reducing respiratory rates (Zhu et al., 2022). However, nighttime warming stimulated the plant’s nighttime respiration, consuming more carbohydrates, thereby promoting compensatory photosynthetic growth the next day (Wan et al., 2009; Turnbull et al., 2002). In this study, nighttime warming did not show the expected photosynthetic compensation effect but significantly inhibited biomass accumulation. This might indicate that the response mechanism of *S. alopecuroides* to nighttime warming differs from that of other species. Additionally, the promoting effect of day-night symmetrical warming on *S. alopecuroides* was consistent with previous findings (Liang et al., 2013), suggesting that climate warming may potentially facilitate the expansion of *S. alopecuroides*. However, direct evidence linking warming-induced growth responses to increased invasive success in natural communities remained limited, and warranted further investigation through competition experiments with native species. Leaves and roots played a key role in resource acquisition and allocation (Lavorel and Grigulis, 2011; Westoby et al., 2002). Plant growth was closely related to resource allocation strategies. In this study, day-night asymmetric warming significantly reduced stem biomass allocation while increasing both leaf and root biomass ratios, though it also reduced specific leaf area (SLA) and root elongation. The decrease in SLA indicated a shift toward thicker, more robust leaves rather than enhanced light capture efficiency per unit biomass, while the reduction in specific root length reflected a transition from extensive to intensive soil resource exploitation. Collectively, these changes suggested that asymmetric warming induced a resource allocation strategy favoring aboveground leaf development and belowground biomass accumulation over stem growth and spatial expansion. This pattern, which prioritized leaf area development and root biomass buildup, might confer competitive advantages under warming conditions (Drenovsky et al., 2012; Funk et al., 2016), although whether this directly translated to enhanced invasiveness required further comparative studies with native species.

Plants responded to oxidative stress by regulating their antioxidant systems, including antioxidant enzymes and antioxidants (Kovacik et al., 2012). This study found that compared with the control, both symmetric and asymmetric warming reduced the soluble protein content of *S. alopecuroides* seedlings, which was consistent with the results of rice under heat stress (Al-Zahrani et al., 2022). Although soluble sugar content did not differ significantly across warming treatments overall, nighttime and symmetric warming both produced lower values than daytime warming. These findings suggested that elevated nighttime temperatures might reduce soluble sugar levels either through enhanced respiratory consumption or via inhibition of photosynthate transport (Bita and Gerats, 2013; Jain et al., 2007). The analysis of antioxidant enzyme system showed that SOD activity increased under all warming treatments, and the asymmetric warming effect was the most significant, which was consistent with findings in rice under heat stress (Al-Zahrani et al., 2022). However, POD activity significantly decreased under asymmetric warming, while CAT activity was significantly lower under daytime warming than that of symmetric warming. The results indicated that different warming patterns might regulate ROS clearance pathways differentially: symmetric warming enhanced CAT activity to clear H_2_O_2_, while asymmetric warming depended on the SOD-GSH pathway (Almeselmani et al., 2006). This differential antioxidant response suggested that *S. alopecuroides* possessed phenotypic plasticity in its stress response mechanisms, which might contribute to its invasive success under variable environmental conditions (Mathakutha et al., 2019; Palma et al., 2021).

The results of this study demonstrated that asymmetric warming, which approximates the observed pattern of climate change in the study region, significantly enhanced seedling growth of *S. alopecuroides* and altered its biomass allocation, as evidenced by increased root biomass accumulation, elevated root-shoot ratio, and enhanced SOD activity. These physiological and morphological adjustments may potentially confer advantages for belowground resource acquisition and environmental stress tolerance, although it should be noted that these inferences were derived from controlled single-species seedling experiments and remain to be validated under field conditions with interspecific competition. These responses are broadly consistent with reports that invasive species often exhibit considerable phenotypic plasticity under environmental changes (Liu et al., 2017a; Hulme, 2017). Combined with previous findings that climate warming can influence plant competitive dynamics (Lu et al., 2013; Stachowicz et al., 2002), our results tentatively suggest that continued asymmetric warming could facilitate the local expansion of *S. alopecuroides* in the Yili grassland, a hypothesis that merits testing through long-term field observations and competition experiments. The increased allocation to root systems under warming conditions may enhance the plant’s capacity for vegetative reproduction via rhizomes (Nosratti et al., 2018); however, the extent to which this physiological response translates into accelerated population spread in natural ecosystems depends on multiple biotic and abiotic factors, including interactions with native competitors, herbivory pressure, and soil resource heterogeneity, that were not addressed in the present study.

## 5 Conclusion

In this study, we systematically explored the trade-off strategies of emergence, seedling growth, resource allocation, and biochemical characteristics of local invasive species *S. alopecuroides* in single warming, symmetric warming, and asymmetric warming environments. The results showed that asymmetric warming significantly altered biomass allocation patterns: root biomass ratio and root-shoot ratio increased, stem biomass allocation decreased, while leaf biomass allocation increased. These changes indicate that asymmetric warming induced a shift toward greater resource allocation to leaf area development and root biomass accumulation at the expense of stem growth. Asymmetric warming significantly increased SOD activity, whereas symmetric warming marginally elevated CAT activity compared with other treatments. These differential antioxidant responses suggest that distinct warming regimes may activate different ROS scavenging pathways in *S. alopecuroides*, reflecting temperature-specific physiological acclimation mechanisms.

Asymmetric warming, as the dominant pattern of climate change in Xinjiang, was associated with altered growth and biomass allocation patterns in this species. The increases in root biomass and root-shoot ratio under asymmetric warming conditions suggest a potential adaptive strategy for enhanced belowground resource acquisition, while the elevated SOD activity indicates activation of protective mechanisms against temperature-induced oxidative stress. Combined with maintained seedling emergence rates and altered leaf development, these physiological and morphological responses provide a plausible mechanism by which climate warming, particularly in its asymmetric form, could contribute to the local expansion of *S. alopecuroides*. Nevertheless, extrapolation from controlled seedling experiments to population-level dynamics in natural ecosystems requires caution, and future studies incorporating interspecific competition and long-term field monitoring are needed to confirm these implications.

In future research on the relationship between global climate change and biological invasion, relevant theoretical models should pay more attention to the key factor of asymmetric warming. At the same time, future research should be combined with long-term climate change predictions to further explore the impact of asymmetric warming on more invasive plants, evaluate their ecological consequences, and accurately predict the spread risk of invasive plants in climate change scenarios. This would help to comprehensively understand the adaptability of invasive plants in changing environments and develop effective management strategies for controlling the spread of *S. alopecuroides* in grassland ecosystems.

## Data Availability

The datasets presented in this study can be found in online repositories. The names of the repository/repositories and accession number(s) can be found below: https://doi.org/10.57760/sciencedb.35763.

## References

[B1] Almeselmani M. Deshmukh P. S. Sairam R. K. Kushwaha S. R. Singh T. P. (2006). Protective role of antioxidant enzymes under high temperature stress. Plant Sci. 171, 382–388. doi: 10.1016/j.plantsci.2006.04.009 22980208

[B2] Alpert P. Bone E. Holzapfel C. (2000). Invasiveness, invasibility and the role of environmental stress in the spread of non-native plants. Perspect. Plant Ecol. Evol. Syst. 3, 52–66. doi: 10.1078/1433-8319-00004

[B3] Alward R. D. (1999). Grassland vegetation changes and nocturnal global warming. Science 283, 229. doi: 10.1126/science.283.5399.229 9880257

[B4] Al-Zahrani H. S. Alharby H. F. Fahad S. (2022). Antioxidative defense system, hormones, and metabolite accumulation in different plant parts of two contrasting rice cultivars as influenced by plant growth regulators under heat stress. Front. Plant Sci. 14, 9111846. doi: 10.3389/fpls.2022.911846 35712584 PMC9196032

[B5] Barton B. T. Schmitz O. J. (2017). Opposite effects of daytime and nighttime warming on top‐down control of plant diversity. Ecology 99, 13–20. doi: 10.1002/ecy.2062 29080358

[B6] Bita C. E. Gerats T. (2013). Plant tolerance to high temperature in a changing environment: scientific fundamentals and production of heat tolerance crops. Front. Plant Sci. 4, 273. doi: 10.3389/fpls.2013.00273 23914193 PMC3728475

[B7] Bradford M. M. (1976). A rapid and sensitive method for the quantitation of microgram quantities of protein utilizing the principle of protein-dye binding. Anal. Biochem. 72, 248–254. doi: 10.1016/0003-2697(76)90527-3 942051

[B8] Chen B. M. Gao Y. Liao H. X. Peng S. L. (2017). Differential responses of invasive and native plants to warming with simulated changes in diurnal temperature ranges. AoB. Plants 9, plx028. doi: 10.1093/aobpla/plx028 28775830 PMC5534020

[B9] Cunze S. Leiblein M. C. Tackenberg O. (2013). Range expansion of Ambrosia artemisiifolia in Europe is promoted by climate change. ISRN. Ecol. 2013, 1–9. doi: 10.1155/2013/610126

[B10] Dhakhwa B. G. Campbell C. L. (1998). Potential effects of differential day-night warming in global climate change on crop production. Clim. Change 40, 647–667. doi: 10.1023/A:1005339800665 41886696

[B11] Drenovsky R. E. Grewell B. J. D'antonio C. M. Funk J. L. James J. J. Molinari N. . (2012). A functional trait perspective on plant invasion. Ann. Bot. 110, 141–143. doi: 10.1093/aob/mcs100 22589328 PMC3380596

[B12] Funk J. L. Standish R. J. Stock W. D. Valladares F. (2016). Plant functional traits of dominant native and invasive species in mediterranean‐climate ecosystems. Ecology 97, 2434–2439. doi: 10.1890/15-0974.1 27008777

[B13] Gao S. Liang E. Liu R. Babst F. Camarero J. J. Fu Y. H. . (2022). An earlier start of the thermal growing season enhances tree growth in cold humid areas but not in dry areas. Nat. Ecol. Evol. 6, 397–404. doi: 10.1038/s41559-022-01668-4 35228669

[B14] He Z. S. He W. M. (2020). Asymmetric climate warming does not benefit plant invaders more than natives. Sci. Tot. Environ. 738, 140262. doi: 10.1016/j.scitotenv.2020.140262 32640393

[B15] Heath R. L. Packer L. (1968). Photoperoxidation in isolated chloroplasts: I. Kinetics and stoichiometry of fatty acid peroxidation. Arch. Biochem. Biophys. 125, 189–198. doi: 10.1016/0003-9861(68)90654-1 5655425

[B16] Hulme P. E. (2017). Climate change and biological invasions: evidence, expectations, and response options. Biol. Rev. 92, 1297–1313. doi: 10.1111/brv.12282 27241717

[B17] Jain M. Prasad P. V. V. Boote K. J. Allen L. H. J. Chourey P. S. (2007). Effects of season-long high temperature growth conditions on sugar-to-starch metabolism in developing microspores of grain sorghum (Sorghum bicolor L. Moench). Planta 227, 67–79. doi: 10.1007/s00425-007-0595-y 17680267

[B18] Jiang X. Jia X. Gao S. Jiang Y. Wei N. Han C. . (2021). Plant nutrient contents rather than physical traits are coordinated between leaves and roots in a desert shrubland. Front. Plant Sci. 12, 734775. doi: 10.3389/fpls.2021.734775 34764966 PMC8576145

[B19] Karl T. R. Knight R. W. Gallo K. P. Peterson T. C. Jones P. D. Kukla G. . (1993). A new perspective on recent global warming: asymmetric trends of daily maximum and minimum temperature. Bull. Am. Meteorol. Soc 74, 1007–1023. doi: 10.1175/1520-0477(1993)074<1007:Anporg>2.0.Co;2

[B20] Kovacik J. Klejdus B. Hedbavny J. Stork F. Gruz J. (2012). Modulation of copper uptake and toxicity by abiotic stresses in Matricaria chamomilla plants. J. Agric. Food. Chem. 60, 6755–6763. doi: 10.1021/jf3013426 22703521

[B21] Lavorel S. Grigulis K. (2011). How fundamental plant functional trait relationships scale‐up to trade‐offs and synergies in ecosystem services. J. Ecol. 100, 128–140. doi: 10.1111/j.1365-2745.2011.01914.x 40046247

[B22] Lei L. J. Zhao Y. Shi K. Liu Y. Hu Y. X. Shao H. (2021). Phytotoxic activity of alkaloids in the desert plant Sophora alopecuroides. Toxins 13, 706. doi: 10.3390/toxins13100706 34678999 PMC8540331

[B23] Liang J. Y. Xia J. Y. Liu L. L. Wan S. Q. (2013). Global patterns of the responses of leaf-level photosynthesis and respiration in terrestrial plants to experimental warming. J. Plant Ecol. 6, 437–447. doi: 10.1093/jpe/rtt003

[B24] Liu Y. Oduor A. M. O. Zhang Z. Manea A. Tooth I. M. Leishman M. R. . (2017a). Do invasive alien plants benefit more from global environmental change than native plants? Glob. Change Biol. 23, 3363–3370. 10.1111/gcb.13579 27888560

[B25] Lu X. Siemann E. Shao X. Wei H. Ding J. (2013). Climate warming affects biological invasions by shifting interactions of plants and herbivores. Glob. Change Biol. 19, 3859–3871. doi: 10.1111/gcb.12244 23640751

[B26] Martinez C. A. Bianconi M. Silva L. Approbato A. Lemos M. Santos L. . (2014). Moderate warming increases PSII performance, antioxidant scavenging systems and biomass production in Stylosanthes capitata Vogel. Environ. Exp. Bot. 102, 58–67. doi: 10.1016/j.envexpbot.2014.02.001 38826717

[B27] Mathakutha R. Steyn C. le Roux P. C. Blonder B. Haider S. Lembrechts J. J. . (2019). Invasive species differ in key functional traits from native and non‐invasive alien plant species. J. Veg. Sci. 30, 178–188. doi: 10.1111/jvs.12772 40046247

[B28] Nosratti I. Amiri S. Bagheri A. Chauhan B. S. (2018). Environmental factors affecting seed germination and seedling emergence of foxtail sophora (Sophora alopecuroides). Weed. Sci. 66, 736–744. doi: 10.1017/wsc.2018.57 41292463

[B29] Palma E. Vesk P. A. White M. Baumgartner J. B. Lai H. R. Robinson K. L. . (2021). Plant functional traits reflect different dimensions of species invasiveness. Ecology 102, e03317. doi: 10.1002/ecy.3317 33638164

[B30] Peng S. Piao S. Ciais P. Myneni R. B. Chen A. Chevallier F. . (2013). Asymmetric effects of daytime and night-time warming on Northern Hemisphere vegetation. Nature 501, 88–92. doi: 10.1038/nature12434 24005415

[B31] Poorter H. Nagel O. (2000). The role of biomass allocation in the growth response of plants to different levels of light, CO2, nutrients and water: a quantitative review. Aust. J. Plant Physiol. 27, 595–607. doi: 10.1071/PP99173 38477348

[B32] Rosenzweig C. Tubiello F. N. (1996). Effects of changes in minimum and maximum temperature on wheat yields in the central US: a simulation study. Agric. For. Meteorol. 80, 215–230. doi: 10.1016/0168-1923(95)02299-6

[B33] Santos R. M. Bakhshoodeh R. (2021). Climate change/global warming/climate emergency versus general climate research: comparative bibliometric trends of publications. Heliyon 7, e08219. doi: 10.1016/j.heliyon.2021.e08219 34765769 PMC8571708

[B34] Shah N. H. Paulsen G. M. (2003). Interaction of drought and high temperature on photosynthesis and grain-filling of wheat. Plant Soil 257, 219–226. doi: 10.1023/a:1026237816578 41886696

[B35] Stachowicz J. J. Terwin J. R. Whitlatch R. B. Osman R. W. (2002). Linking climate change and biological invasions: ocean warming facilitates nonindigenous species invasions. Proc. Natl. Acad. Sci. U.S.A. 99, 15497–15500. doi: 10.1073/pnas.242437499 12422019 PMC137745

[B36] Turnbull M. H. Murthy R. Griffin K. L. (2002). The relative impacts of daytime and nighttime warming on photosynthetic capacity in Populus deltoides. Plant Cell Environ. 25, 1729–1737. doi: 10.1046/j.1365-3040.2002.00947.x 37945311

[B37] Wan S. Q. Xia J. Y. Li W. X. Niu S. L. (2009). Photosynthetic overcompensation under nocturnal warming enhances grassland carbon sequestration. Ecology 90, 2700–2710. doi: 10.1890/08-2026.1 19886480

[B38] Wang Z. B. Yang H. J. Dong B. Q. Zhou M. M. Ma L. Y. Jia Z. K. . (2017). Effects of canopy gap size on growth and spatial patterns of Chinese pine (Pinus tabulaeformis) regeneration. For. Ecol. Manage. 385, 46–56. doi: 10.1016/j.foreco.2016.11.022 38826717

[B39] Wang J. Zhang Q. Song J. Ru J. Zhou Z. Xia J. . (2020). Nighttime warming enhances ecosystem carbon‐use efficiency in a temperate steppe. Funct. Ecol. 34, 1721–1730. doi: 10.1111/1365-2435.13579 40046247

[B40] Wei B. Zhang D. Wang G. Liu Y. Li Q. Zheng Z. . (2023). Experimental warming altered plant functional traits and their coordination in a permafrost ecosystem. New Phytol. 238, 232–245. doi: 10.1111/nph.19115 37434301

[B41] Westoby M. Falster D. S. Moles A. T. Vesk P. A. Wright I. J. (2002). Plant ecological strategies: some leading dimensions of variation between species. Annu. Rev. Ecol. Syst. 33, 125–159. doi: 10.1146/annurev.ecolsys.33.010802.150452 41139587

[B42] Wu H. Ismail M. Ding J. (2017). Global warming increases the interspecific competitiveness of the invasive plant alligator weed, Alternanthera philoxeroides. Sci. Tot. Environ. 575, 1415–1422. doi: 10.1016/j.scitotenv.2016.09.226 27720597

[B43] Xia H. Li A. Feng G. Li Y. Qin Y. Lei G. . (2018). The effects of asymmetric diurnal warming on vegetation growth of the Tibetan Plateau over the past three decades. Sustainability 10, 1103. doi: 10.3390/su10041103 30654563

[B44] Yemm E. W. Willis A. J. (1954). The estimation of carbohydrates in plant extracts by anthrone. Biochem. J. 57, 508–514. doi: 10.1042/bj0570508 13181867 PMC1269789

[B45] Yuan J. Yu X. Wu T. Gao S. Zhang T. Yan Q. . (2025). Asymmetric warming of day and night benefits the early growth of Acer mono seedlings more than symmetric warming. Plant Cell Environ. 48, 272–285. doi: 10.1111/pce.15127 39253998

[B46] Zhao J. Liu X. J. Du Z. Q. Wu Z. T. (2017). Effects of the asymmetric diurnal-warming on vegetation dynamics in Xinjiang. China Environ. Sci. 37, 2316–2321. doi: 10.3969/j.issn.1000-6923.2017.06.019

[B47] Zhong Z. He B. Chen H. W. Chen D. Zhou T. Dong W. . (2023). Reversed asymmetric warming of sub-diurnal temperature over land during recent decades. Nat. Commun. 14, 7189. doi: 10.1038/s41467-023-43007-6 37938565 PMC10632450

[B48] Zhu G. Wang X. Xiao J. Zhang K. Wang Y. He H. . (2022). Daytime and nighttime warming has no opposite effects on vegetation phenology and productivity in the northern hemisphere. Sci. Tot. Environ. 822, 153386. doi: 10.1016/j.scitotenv.2022.153386 35093352

